# Dietary Acid Load but Not Mediterranean Diet Adherence Score Is Associated With Metabolic and Cardiovascular Health State: A Population Observational Study From Northern Italy

**DOI:** 10.3389/fnut.2022.828587

**Published:** 2022-04-26

**Authors:** Juana Maria Sanz, Domenico Sergi, Simona Colombari, Eleonora Capatti, Roberta Situlin, Gianni Biolo, Filippo Giorgio Di Girolamo, Stefano Lazzer, Boštjan Šimunič, Rado Pišot, Angelina Passaro

**Affiliations:** ^1^Department of Chemical and Pharmaceutical and Agricultural Sciences, University of Ferrara, Ferrara, Italy; ^2^Department of Translational Medicine, University of Ferrara, Ferrara, Italy; ^3^University Hospital of Ferrara Arcispedale Sant'Anna, Ferrara, Italy; ^4^Department of Medical Surgical and Health Sciences, Clinica Medica ASUGI, University of Trieste, Trieste, Italy; ^5^Hospital Pharmacy, Cattinara Hospital, Azienda Sanitaria Universitaria Giuliano Isontina, Trieste, Italy; ^6^Department of Medicine, University of Udine, Udine, Italy; ^7^Institute for Kinesiology Research, Science and Research Center of Koper, Koper, Slovenia

**Keywords:** Mediterranean diet, dietary acid load, PRAL, NEAP, alkaline diet, acidic diet, metabolic syndrome, cardiovascular risk score

## Abstract

Diet plays a pivotal role in shaping the trajectory of chronic diseases. In this regard, the Mediterranean diet has been widely shown to exert beneficial effects on cardiometabolic health. On the contrary, the Western diet, which has also been reported to be an acidogenic dietary pattern, elicits detrimental effects on both metabolic and cardiovascular (CV) health. However, the role of dietary acid load (DAL) as a predictor of cardiometabolic prognosis remains to be elucidated. Thus, this study aims to compare Mediterranean diet adherence (MDA) and DAL focusing on their relationship with metabolic and CV prognosis. A total of 448 individuals aged 55–80 years were grouped depending on their MDA, assessed using food frequency questionnaires, or DAL, evaluated using potential renal load acid (PRAL) and net-endogenous acid production (NEAP). Study participants underwent anthropometric and biochemical measurements. The metabolic syndrome (MetS) prevalence was evaluated according to the National Cholesterol Education Program-Adult Treatment Panel III. Finally, the CV risk was evaluated using three independent algorithms: atherosclerotic cardiovascular disease (ASCVD), European Systematic COronary Risk Evaluation (SCORE), and Cuore risk scores. Mediterranean diet adherence was negatively associated with PRAL and NEAP. Individuals in the higher MDA tertile group had higher HDL cholesterol as well as lower homeostasis model assessment index (HOMA-IR) and fat mass relative to the lowest MDA tertile. However, in the high-MDA tertile group, there was neither a significantly lower MetS prevalence nor CV risk. Instead, both the MetS prevalence and CV risk were higher in individuals in the higher acid PRAL quartile relative to the lower alkaline PRAL quartile. Dietary acid load, especially assessed using PRAL but not MDA, was associated with indices of metabolic and CV prognosis. Thus, DAL assessed by 24-h dietary recalls may represent a better predictor of cardiometabolic health if compared to MDA evaluated using food frequency questionnaires.

## Introduction

Obesity poses a huge threat to human health as well as a burden to the healthcare systems worldwide (1, 2). In fact, obesity is a key risk factor for the development of metabolic syndrome (MetS), a constellation of cardiometabolic risk factors that include central obesity, impaired glucose tolerance and insulin resistance, dyslipidemia, and hypertension (3). Unhealthy dietary patterns, alongside physical inactivity, represent the primary environmental factors to blame for the ramping up of the obesity epidemic and its cardiometabolic comorbidities. In fact, the consumption of long-chain saturated fatty acids, sugar, and processed foods, typical of the Western diet, has been widely reported to impair cardiometabolic health by fostering obesity, insulin resistance, and cardiovascular (CV) disease (4–6). On the contrary, the Mediterranean diet (MD) exerts a beneficial effect on metabolic as well as CV health (7, 8). This is because this dietary pattern is characterized by a wide consumption of plant foods (fruit, vegetables, legumes, nuts and seeds, cereals, and preferably wholegrain); the choice of seasonal, fresh, and locally grown products; the use of olive oil as the main source of added lipids; low to moderate amounts of dairy products (mainly low-fat), poultry, fish, and eggs; and a moderate intake of red wine and limited consumption of red meat and sugar (9). In concomitance with the abundance of these food groups, this dietary pattern is also abundant in a variety of food bioactives, including polyphenols, monounsaturated and polyunsaturated fatty acids, whose combinations contribute to the health-promoting effects of the MD (10).

Despite the well-documented effects of the MD on health, the adherence to this dietary pattern is declining (11) in favor of the Western diet, even in the Mediterranean area. The Western diet is a dietary pattern characterized by increased consumption of sugary drinks, red meat, highly processed foods, high-glycemic-index carbohydrates, and long-chain saturated fatty acids (12), which, as already mentioned, are metabolically detrimental. This trend is accompanied by a rise in the acid load of the diet due to an increase in protein intake, which is paralleled by a concomitant decrease in the consumption of alkalis derived from fruits and vegetables. Dietary acid load (DAL) can be computed by using different algorithms, such as potential renal acid load (PRAL) based on dietary protein, phosphorus, potassium, calcium, and magnesium intake and net-endogenous acid production (NEAP) based on protein and potassium intake.

Low and negative PRAL as opposed to high (positive) PRAL are the results of “alkaline and acidic diet,” respectively. The concept that an acidic diet is associated with negative health outcomes is rampant among the public, but scientific evidence in this context remains to be consolidated and is mainly limited to the effect of DAL on bone mass and kidney stones. In fact, the effects of an acidic diet (with high PRAL values) on pathological conditions, such as hypertension, diabetes, and CV disease, are yet to be fully elucidated (13, 14) and remain a matter of contention (15–17). While the MD has been widely demonstrated to elicit positive effects on cardiometabolic health (18–20) and may decrease DAL, it remains to identify a putative association between the MD and DAL, assessed using PRAL or NEAP. At present, there is no data in the literature that compare the impact of MDA and DAL on metabolic and CV health.

Thus, the aim of this study is to investigate the relationship between MDA or DAL and indices of metabolic and CV prognosis, such as the prevalence of MetS and CV risk in an Italian adult population.

## Subjects and Methods

### Participants

A total of 459 free-living individuals aged 55–80 years and able to walk for 2 km without any aids were enrolled in the Physical Activity and Nutrition for Quality Aging (PANGeA) study in Gemona, Trieste, and Ferrara between 2013 and 2014 (https://ec.europa.eu/regional_policy/en/projects/italy/pangea-keeping-an-aging-population-moving). Subjects having cancer, a history of hospitalization in the last 12 months, or who were taking anticoagulants were excluded. PANGEeA's participants with missing food frequency questionnaire were excluded from this study (*N* = 2.4%; Supplementary Figure 1). The remaining 448 subjects were clinically evaluated through interviews and physical examinations and underwent blood sampling and anthropometric measurements. The characteristics of the study population are reported in Table 1.

**Table 1:** Characteristics of the study population.

	**Media ±SD Median (95% CI) Number (%)**
Subjects, number	448
Age, years	66.6 ± 4.7
Sex Female, number (%)	248 (55.4)
Smoke, number (%)	41 (9.2)
BMI (kg/mq)	26.6 ± 3.8
Cardiovascular disease, number (%)	19 (4.3)
Hypertension, number (%)	135 (30.1)
Diabetes Mellitus, number (%)	23 (5.1)
Metabolic syndrome, number (%)	72 (16.1)
History cancer, number (%)	31 (6.9)
SBP (mmHg)	139 ± 19
DBP (mmHg)	85 ± 10
MDA score	17.0 (13.0–21.0)
PRAL (mEq/day)	6.6 (-23.7–36.6)
NEAP (mEq/day)	45.9 (21.7–78.8)

*SD, standard deviation; CI, confidence interval; BMI, body mass index; MDA, Mediterranean diet adherence; SBP, systolic blood pressure; DBP, diastolic blood pressure; PRAL, potential renal acid load; NEAP, net-endogenous acid production*.

Written informed consent was obtained from each patient with *no personal information being available to the authors (blinding)*. Strengthening the reporting of observational studies in epidemiology (STROBE) guidelines were followed to report observational data as well as for the preparation of this manuscript. To decrease the risk of selection bias, study participants were consecutively recruited and included in the study. Furthermore, to decrease the risk of bias, trained specific investigators were designed for the assessment of each outcome independently of the recruitment center.

This study was approved by the National Ethical Committee of the Slovenian Ministry of Health on 17 April 2012, under the acronym IR-aging 1200, and it conformed to the ethical principles for medical research involving human subjects as required by the 2013 Review of the Helsinki Declaration of Helsinki—Ethical Principles for Medical Research Involving Human Subjects.

### Dietary Assessment

#### Assessment of Mediterranean Diet Adherence

Mediterranean diet adherence was based on a food frequency questionnaire administered to study participants by trained interviewers (nutrition expert medical doctors). The food frequency questionnaire encompassed a 90-item food and beverage list and allowed participants to indicate the consumption frequency of these items as follows: multiple times/day; 1 time/day; 5–6 times/week; 2–4 times/week; 1 time/week; 1–3 times/month, and never. To assess MDA, the consumption frequency of the following 13 main food categories was taken into consideration: milk and dairy products, cereals and grain products, vegetables, legumes, fruits, olive oil, white meat, red and processed meat, fish, sweets and desserts, nuts and seeds, and wine. Scores of zero, one, or two points indicated a low, medium, or high adherence to the MD pyramid, respectively (Supplementary Table 1). The MDA was the sum of single category scores (MDA range: 0–26 points). Scores were based on the dietary guidelines of the MD (9).

#### 24-H Recall and DAL

Nutritional assessment was conducted through two repeated 24-h dietary recalls, which is a retrospective and quantitative method to gather information about foods and beverages consumed by the participants in 24 h prior to the visit. Two recalls were collected by trained interviewers as follows: the first one personally on the day of the visit and the second one after 2 months over the phone. Data from 24-h recall were analyzed using the nutrient analysis software Winfood® PRO 3.9.x (Medimatica Surl, Teramo, Italy) to obtain total energy and macro and micronutrients intake for each individual interview. Results were the average of the two 24-h recalls.

Data relative to nutrient intake were used to determine DAL by two algorithms, yielding PRAL (21) and NEAP (22).


(1) $$\begin{array}{r} PRAL\left(\frac{\text{mEq}}{\text{d}}\right)=0.4888\cdot \text{protein}\left(\frac{\text{g}}{\text{d}}\right)\\ \;+0.0366\cdot \text{phosphorus}\left(\frac{\text{mg}}{\text{d}}\right)\\ \;-0.0205\cdot \text{potassium }\left(\frac{\text{mg}}{\text{d}}\right)\\ \;-0.0125\cdot \text{calcium}\left(\frac{\text{mg}}{\text{d}}\right)\\ \;-0.0263\cdot \text{magnesium}\left(\frac{\text{mg}}{\text{d}}\right) \end{array}$$

(2) $$\begin{array}{r} \text{NEAP }\left(\frac{\text{mEq}}{\text{d}}\right)=54.5\cdot \frac{\text{protein}\left(\frac{\text{g}}{\text{d}}\right)}{\text{potassium}\left(\frac{\text{mEq}}{\text{d}}\right)}-10.2 \end{array}$$

### Anthropometric Measurements

Anthropometric characteristics were evaluated in participants wearing light clothing with no restrictive underwear and no shoes.

Anthropometric measures included body mass index (BMI); body weight rounded to the nearest 100 g; height, waist, and hip circumferences all rounded to the nearest 0.1 cm; waist circumference measured around the smallest circumference between the lowest rib and iliac crest; hip circumference measured horizontally at the level of the greatest lateral extension of the hips.

### Bioelectrical Impedance Analysis (BIA)

Body composition (total body water, fat mass, free fat mass, muscle cells, and body cell mass) and basal metabolic rate were estimated by the same trained staff member, using bioimpedance with a tetrapolar impedance meter (BIA101, Akern, Florence, Italy) according to the manufacturer's instructions (23). All measures were conducted with the patient lying down, after 8 h fasting.

### Biochemical Analysis

Blood samples were collected after an overnight fast and centrifuged at 1,600 × *g* for 15 min at 4°C to obtain serum or plasma. Samples were aliquoted and stored at 80°C until use.

Total cholesterol, HDL cholesterol, triglycerides, glucose, and insulin were assayed using standard enzymatic-colorimetric methods (24). LDL cholesterol was calculated using Friedewald's formula (25). Insulin resistance was assessed using the homeostasis model assessment index (HOMA-IR) which was computed as follows (25):


(3) $$\begin{array}{r} HOMA-IR\;index=\frac{glucose\left(\frac{mmol}{l}\right)\cdot insulin\left(\frac{mU}{l}\right)}{22.5} \end{array}$$

### MetS Score

Metabolic syndrome was defined according to the National Cholesterol Education Program-Adult Treatment Panel III (NCEP ATP III) and diagnosed in the presence of three or more of the following five criteria: (1) waist circumference ≥102 cm in men or ≥88 cm in women; (2) the use of antihypertensive medications, systolic blood pressure ≥130 mmHg or diastolic blood pressure ≥85 mmHg; (3) fasting triglycerides level ≥ 150 mg/dl or taking antihyperlipidaemic drugs; (4) fasting HDL cholesterol ≤ 40 mg/dl in men or ≤ 50 mg/dl in women or pharmacological treatment for low HDL cholesterol; and (5) fasting blood glucose ≥110 mg/dl or taking hypoglycaemic medications (26). MetS score ranged from 0 to 5 depending on the number of positive criteria.

### Evaluation of CV Risk

The probability of having a major CV event in the 10 years post assessment was estimated for study participants without a history of CV disease (*N* = 429), using the following major CV risks scores: (a) atherosclerotic cardiovascular disease (ASCVD) risk score developed by the American College of Cardiology/American Heart Association Atherosclerotic Cardiovascular Disease (ACC/AHA ASCVD) and applicable to individuals aged 40–79 years (27); (b) European Systematic COronary Risk Evaluation (SCORE) developed by the European Society of Cardiology and applicable to individuals aged 45–64 years (28). The number of participants of this study with an age below 65 was 203; given the considerable impact on the participant number, the score was calculated regardless of age; and (c) Cuore risk score (Progetto CUORE individual score, National Institute of Health, Italy), based on Italian epidemiological data and applicable to individuals aged 35–69 years (29, 30).

The variables included in the calculation of those scores were age, gender, current smoking habit, TC, HDL-C (except for the European SCORE), systolic blood pressure, diagnosis of hypertension or pharmacological treatment of hypertension, and diagnosis of diabetes or taking hypoglycemic medications (except for the European SCORE). Participants with a history of CV events were excluded from risk estimation (*N* = 19).

### Statistical Analysis

Continuous variables were analyzed for normal distribution using Shapiro–Wilk tests and expressed as mean ± standard deviation (SD) or median (95% confidence interval, CI) for normally and non-normally distributed variables, respectively. One-way ANOVA or Kruskal–Wallis tests were used to assess overall differences between groups, and Dunnet or Mann–Whitney tests were performed for comparisons between extreme tertiles or quartiles (high MDA vs. low MDA and strong PRAL vs. alkaline PRAL). Categorical variables were compared with exact Fisher or chi-squared tests. Spearman's correlation coefficient was used to test the association between MDA, PRAL, or NEAP and the parameters of interest. A *p*-value of ≤ 0.05 was considered statistically significant.

Missing data for each variable of interest did not exceed 5%.

## Results

### Study Population and Dietary Assessment

Assessment of MDA revealed that only 20 individuals included in the study (4.5%) had a score lower than 13 points (the arithmetic means of the 0–26 MDA range), while half of the study participants had an MDA score ranging between 15 and 18 points (Table 1). The scores of the 13 main food categories used to calculate the MDA score are reported in Supplementary Table 1. Compliance with the guidelines set out in the MD pyramid was higher for the consumption of vegetables, milk and dairy products, wheat, and fruit (Supplementary Table 2). Instead, the lowest compliance was observed relative to the consumption of nuts and sweets. Another poorly followed MD recommendation concerned the consumption of processed and red meat. The minimum score (zero) for the consumption of these foods was obtained by 176 participants (39.3%), of which 6 (1.3%) almost never ate red or processed meat, while the other 169 (37.7%) ate it more than 4 times a week.

Correlation analysis between MDA score, PRAL, and NEAP as well as energy, micronutrient, and macronutrient intake is depicted in Table 2. MDA correlated negatively with PRAL and NEAP. There was no association between the total energy intake and MDA. Instead, greater adherence to MD was positively associated with the intake of total dietary fiber; oligosaccharides; microelements such as calcium, potassium, phosphorous, iron, and zinc; and vitamins such as riboflavin, thiamine, vitamin C, and vitamin E. Furthermore, MDA was correlated positively with alcohol intake, which is in agreement with the fact that the mild and regular consumption of wine at mealtimes is one of the cornerstones of the MD.

**Table 2:** Correlation between MDA score, PRAL or NEAP, and dietary parameters.

	**MDA**		**PRAL (mEq/day)**		**NEAP (mEq/day)**	
	**r_**S**_**	***p*-value**	**r_**S**_**	***p*-value**	**r_**S**_**	***p*-value**
MDA score			−0.122^**^	0.010	−0.158^**^	0.001
PRAL (mEq/day)	−0.122^**^	0.010			0.859^**^	0.000
NEAP (mEq/day)	−0.158^**^	0.001	0.859^**^	0.000		
Total calories (kcal/day)	0.032	0.500	0.243^**^	0.000	0.156^**^	0.001
Alcol (kcal/day)	0.179^**^	0.000	0.050	0.290	0.008	0.863
Protein (g/day)	0.078	0.102	0.413^**^	0.000	0.375^**^	0.000
Lipid (g/day)	−0.001	0.988	0.202^**^	0.000	0.170^**^	0.000
Carbohydrates (g/day)	0.013	0.786	0.085	0.072	−0.002	0.969
Starch (g/day)	−0.097^*^	0.041	0.158^**^	0.001	0.127^**^	0.007
Oligosaccharides (g/day)	0.174^**^	0.000	−0.253^**^	0.000	−0.324^**^	0.000
Total fiber (g/day)	0.216^**^	0.000	−0.272^**^	0.000	−0.336^**^	0.000
Cholesterol (mg/day)	0.040	0.403	0.320^**^	0.000	0.241^**^	0.000
Saturated fatty acids (g/day)	−0.033	0.482	0.258^**^	0.000	0.204^**^	0.000
PUFAs (g/day)	0.044	0.351	0.101^*^	0.032	0.015	0.757
MUFAs (g/day)	0.074	0.120	0.117^*^	0.014	0.069	0.146
Calcium (mg/day)	0.140^**^	0.003	0.069	0.146	0.030	0.522
Sodium (mg/day)	−0.041	0.383	0.169^**^	0.000	0.142^**^	0.003
Potassium (mg/day)	0.195^**^	0.000	−0.381^**^	0.000	−0.518^**^	0.000
Phosphorus (mg/day)	0.107^*^	0.024	0.412^**^	0.000	0.119^*^	0.012
Magnesium (mg/day)	0.046	0.605	0.224	0.791	−0.110	0.217
Iron (mg/day)	0.120^*^	0.011	0.032	0.497	−0.056	0.237
Zinc (mg/day)	0.094^*^	0.046	0.081	0.086	−0.031	0.513
Folic acid (mcg/day)	0.082	0.084	−0.186^**^	0.000	−0.309^**^	0.000
Niacin (mg/day)	0.041	0.384	0.173^**^	0.000	0.017	0.725
Riboflavin (mg/day)	0.120^*^	0.011	0.044	0.356	−0.052	0.276
Tiamin (mg/day)	0.087	0.067	−0.052	0.271	−0.129^**^	0.006
Vitamin A (mcg/day)	0.094^*^	0.048	−0.165^**^	0.000	−0.232^**^	0.000
Vitamin B6 (mg/day)	0.044	0.364	−0.101^*^	0.037	−0.257^**^	0.000
Vitamin C (mg/day)	0.206^**^	0.000	−0.473^**^	0.000	−0.528^**^	0.000
Vitamin D (mg/day)	0.006	0.892	0.120^*^	0.011	0.020	0.669
Vitamin E (mg/day)	0.163^**^	0.001	−0.168^**^	0.000	−0.255^**^	0.000

*MDA, Mediterranean diet adherence; PRAL, potential renal acid load; NEAP, net-endogenous acid production; rs, Spearman correlation coefficient; PUFAs, polyunsaturated fatty acids; MUFAs, monounsaturated fatty acids*.

* *p-value < 0.05*;

** *p-value < 0.01*.

As expected, there was a strong positive and statistically significant correlation between PRAL and NEAP. These DAL-related parameters showed a positive association with the intake of total calories, protein, starch, lipid (total cholesterol and saturated lipid), sodium, and phosphorous. On the contrary, an increase in DAL was associated with a decrease in the intake of oligosaccharides, total dietary fiber, potassium, and some vitamins (folic acid, thiamine, vitamin B6, vitamin C, vitamin A, and vitamin E). Despite both PRAL and NEAP being related to DAL, there were differences between these DAL proxies. Only PRAL was positively correlated with monounsaturated and polyunsaturated fatty acids, niacin, and vitamin D intake, while only NEAP was negatively correlated with tiamin (Table 2).

### Anthropometrics, Body Composition, and Metabolic Parameters

To investigate the effects of dietary habits on selected health outcomes, PRAL was divided into quartiles to associate this parameter with an alkaline, neutral, slightly acidic, or strongly acidic diet (alkaline PRAL, neutral PRAL, light PRAL, and strong PRAL, respectively). Similar to PRAL, NEAP was divided into quartiles. Instead, the MDA score range of the study participants was too tight to separate them into quartiles; therefore, they were divided into tertiles as follows: low MDA, medium MDA, and high MDA.

Participants in the low-MDA tertile, relative to individuals in the high-MDA tertiles, had a higher BMI and fat mass; a worse metabolic profile marked by higher triglyceride concentrations, lower HDL cholesterol, and higher HOMA-IR values. Furthermore, study participants in the low-MDA tertile had a higher DAL, as assessed by NEAP, compared to participants in the high-MDA tertile (Table 3). Despite the rest of the parameters analyzed did not reach statistical significance between MDA tertiles, the differences in waist circumference, triglycerides, and PRAL tended to be significant when comparing individuals in the low- and high-MDA tertiles (*p* = 0.052, *p* = 0.069, and *p* = 0.056, respectively).

**Table 3:** Population characteristics per MDA score tertiles.

	**Low-MDA**	**Medium-MDA**	**High-MDA**	**^#^*p*-value**	**^§^*p*-value**
**N**	136	137	173		
MDA score	14.0 (11.0–15.0)	17.0 (16.0–17.0)	19.0 (18.0–22.3)		
PRAL (mEq/day)	10.0 (-24.8–43.2)	8.2 (-19.4–36.8)	4.5 (-25.6–33.6)	0.152	0.056
NEAP (mEq/day)	48.5 (20.5–84.7)	45.0 (23.8–75.2)	42.7 (20.8–78.6)	0.025^*^	0.008^**^
Age (years)	66.0 (60.0–74.2)	65.0 (61.0–75.0)	66.0 (60.0–76.0)	0.174	0.092
SBP (mmHg)	136.0 (112.7–173.0)	138.2 (111.6–178.0)	135.7 (107.0–171.5)	0.326	0.408
DBP (mmHg)	86.2 (73.3–100.4)	83.7 (66.9–104.0)	83.3 (69.2–102.0)	0.324	0.127
TG (mg/dL)	97.0 (55.2–208.4)	89.0 (45.0–168.4)	90.0 (49.3–179.0)	0.072	0.069
Total-C (mg/dL)	218.1 ± 39.9	213.2 ± 38.6	218.2 ± 36.8	0.470	1.000
LDL-C (mg/dL)	133.4 ± 32.6	125.3 ± 35.7	130.5 ± 32.8	0.145	0.698
HDL-C (mg/dl)	62.8 ± 15.4	68.8 ± 18.1	68.4 ± 17.9	0.005^**^	0.010^*^
Glucose (mg/dL)	95.0 (80.8–142.6)	98.0 (76.0–126.6)	97.0 (80.0–123.6)	0.762	0.771
Insulin (mU/L)	8.6 (3.8–23.7)	7.8 (3.6–19.2)	7.5 (3.4–19.4)	0.043^*^	0.013^*^
HOMA-IR	2.0 (0.8–6.5)	1.8 (0.8–5.5)	1.8 (0.8–5.5)	0.074	0.027^*^
BMI (kg/mq)	26.9 (21.3–34.3)	25.8 (21.5–34.1)	26.1 (20.7–32.8)	0.038^*^	0.014^*^
Waist circ. (cm)	93.0 (76.7–112.2)	92.0 (75.9–113.0)	91.0 (73.6–110.9)	0.139	0.052
BMR (Kcal/day)	1,400.5 (1211.1–1705.0)	1,448.0 (1225.3–1664.9)	1,358.0 (1218.8–1675.8)	0.266	0.220
Free Fat Mass (%)	64.4 (53.1–74.0)	64.7 (52.1–76.2)	64.9 (54.5–74.4)	0.306	0.132
Free Fat Mass (kg)	45.9 (34.6–66.2)	45.6 (34.9–61.9)	43.0 (34.3–62.2)	0.289	0.234
Fat Mass (%)	35.6 (26.0–46.9)	35.3 (23.8–47.9)	35.1 (25.6–45.5)	0.306	0.132
Fat Mass (kg)	26.0 (17.3–39.9)	24.5 (15.7–41.6)	23.4 (15.0–37.9)	0.030^*^	0.008^**^
Total body water (L)	35.9 (27.7–50.1)	36.1 (27.9–49.5)	34.1 (27.5–49.6)	0.286	0.265
Muscle Mass (kg)	28.2 (20.4–41.0)	29.1 (20.8–39.6)	26.8 (20.5–39.9)	0.339	0.217
Body Cell Mass (kg)	22.7 (16.2–33.2)	23.7 (16.4–32.7)	21.0 (16.2–32.0)	0.263	0.182

*Data are expressed as mean ±SD or median (95% CI)*.

# *p-value is based on ANOVA or Kruskal–Wallis test to compare the three MDA subgroups*.

§ *p-value is based on Dunnet or Mann–Whitney test to compare low MDA against high MDA. SD, standard deviation; CI, confidence interval; MDA, Mediterranean diet adherence; PRAL, potential renal acid load; NEAP, net-endogenous acid production; SBP, systolic blood pressure; DBP, diastolic blood pressure; TG, triglycerides; Total-C, total cholesterol; LDL-C, low-density lipoprotein cholesterol; HDL-C, high-density lipoprotein cholesterol; HOMA-IR, homeostatic model assessment for insulin resistance; BMI, body mass index; Waist-circ, waist circumference; BMR, basal metabolic rate*.

* *p-value < 0.05*;

** *p-value < 0.01*.

When DAL was used to compare the metabolic and anthropometric characteristics of the study participants, individuals in the highest PRAL quartile (strong PRAL) (Table 4) were characterized by lower total, LDL, and HDL cholesterol concentrations; higher waist circumference; free fat mass; glucose concentration; and HOMA-IR (Table 4). Anthropometric data analysis revealed higher basal metabolic rate, muscle cell, and body cell mass in strong PRAL vs. alkaline PRAL.

**Table 4:** Population characteristics per PRAL quartile.

	**Alkaline-PRAL**	**Neutral-PRAL**	**Light-PRAL**	**Strong-PRAL**	**^#^*p*-value**	**^§^*p*-value**
N	111	113	112	112		
MDA- score	17.0 (13.0–21.4)	17.0 (13.0–21.3)	17.0 (12.7–20.0)	16.0 (12.0 −21.0)	0.036^*^	0.008^**^
PRAL	−13.6 (-36.6–4.0)–	1.8 (-3.2–6.1)	12.8 (7.9–17.2)	28.1 (18.3–62.7)		
NEAP	28.8 (16.6–41.3)	40.7 (30.9–54.9)	51.4 (39.1–78.4)	64.4 (42.1–104.6)	<0.001^***^	<0.001^***^
Age (years)	65.0 (60.0–75.0)	66.0 (60.0–75.0)	66.0 (60.0–76.4)	66.0 (60.0–75.0)	0.732	0.900
SBP (mmHg)	137,3 (106,9–169,4)	136,3 (112,4–170,3)	137,5 (107,0–179,4)	135,7 (112,9–177,4)	0.834	0.683
DBP (mmHg)	85,0 (67,2–102,0)	87,0 (69,8–100,5)	83,3 (68,4–104,0)	83,8 (72,1–103,4)	0.643	0.887
TG (mg/dL)	87,5 (53,0–171,2)	96,5 (46,4–180,3)	92,5 (53,6–179,0)	93,0 (53,4–199,8)	0.683	0.325
Total-C (mg/dL)	224.2 ± 38.4	221.6 ± 39.8	214.0 ± 36.6	206.9 ± 36.2	0.003^**^	0.002^**^
LDL-C (mg/dL)	134.3 ± 35.5	134.3 ± 35.5	128.0 ± 30.0	122.5 ± 32.6	0.026^*^	0.027^*^
HDL-C (mg/dl)	70.4 ± 17.0	67.3 ± 19.4	66.4 ± 16.7	63.3 ± 15.7	0.027^*^	0.008^**^
Glucose (mg/dL)	95.0 (76.8–117.6)	97.0 (81.4–133.6)	99.0 (82.5–122.5)	104.7 ± 28.3	0.011^*^	0.002^**^
Insulin (mU/L)	7.7 (3.5–18.2)	7.3 (4.3–19.0)	8.6 (3.2–21.2)	8.2 (3.3–25.0)	0.618	0.406
HOMA IR	1.8 (0.8–4.9)	1.7 (1.0–6.2)	2.1 (0.8–5.9)	2.1 (0.7–7.3)	0.057	0.020^*^
BMI (kg/mq)	26.0 (20.8–33.6)	26.0 (21.2–32.9)	26.3 (21.4–33.7)	26.5 (21.2–34.5)	0.743	0.770
Waist circ. (cm)	90.0 (71.6–108.0)	92.0 (75.8–109.3)	94.0 (77.7–112.4)	92.0 (77.0–115.4)	0.011^*^	0.010^*^
BMR (Kcal/die)	1,336.0 (1212.0–1642.6)	1,390.0 (1227.5–1680.9)	1,384.0 (1216.6–1746.6)	1,458.4 (1213.9–1676.1)	0.015^*^	0.004^**^
Free fat mass (%)	64.3 (52.0–74.7)	65.9 (52.2–76.1)	63.8 (53.7–77.1)	65.9 (53.9–74.1)	0.553	0.464
Free fat mass (kg)	41.9 (34.5–60.6)	43.8 (34.6–62.5)	44.8 (34.7–62.6)	49.7 (34.0–62.9)	0.015^*^	0.004^**^
Fat mass (%)	35.7 (25.3–48.0)	34.1 (23.9–47.8)	36.2 (22.9–46.3)	34.2 (25.9–46.1)	0.553	0.464
Fat mass (kg)	24.8 (15.7–40.0)	23.4 (15.7–38.6)	24.6 (14.0–38.8)	25.3 (16.7–42.1)	0.545	0.651
Total body water (L)	32.8 (27.6–48.4)	34.7 (27.7–50.0)	35.5 (27.8–50.1)	39.4 (27.2–49.6)	0.020^*^	0.005^**^
Muscle mass (kg)	25.6 (20.5–38.6)	27.5 (20.6–40.0)	27.2 (20.5–42.5)	30.7 (20.4–40.0)	0.021^*^	0.006^**^
Body cell Mass (kg)	20.4 (16.3–30.9)	22.1 (16.3–32.3)	21.6 (16.1–34.5)	24.5 (16.0–32.0)	0.026^*^	0.002^**^

*Data are expressed as mean ±SD or median (95% CI)*.

# *p-value is based on ANOVA or Kruskal–Wallis test to compare the four PRAL subgroups*.

§ *p-value is based on Dunnet or Mann–Whitney test to compare strong PRAL against basic PRAL. SD, standard deviation; CI, confidence interval; MDA, Mediterranean diet adherence; PRAL, potential renal acid load; NEAP, net-endogenous acid production; SBP, systolic blood pressure; DBP, diastolic blood pressure; TG, triglycerides; Total-C, total cholesterol; LDL-C, low-density lipoprotein cholesterol; HDL-C, high-density lipoprotein cholesterol; HOMA-IR, homeostatic model assessment for insulin resistance; BMI, body mass index; Waist-circ., waist circumference; BMR, basal metabolic rate*.

* *p-value < 0.05*;

** *p-value < 0.01*;

*** *p-value < 0.001*.

### MetS Score

**Figure 1:**
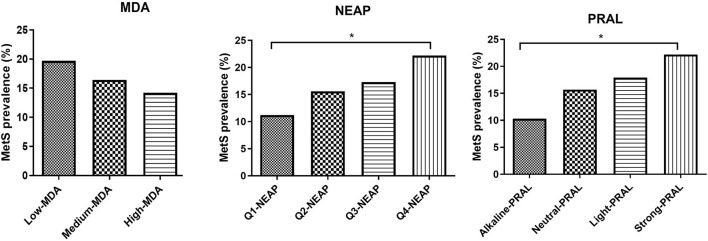
MetS prevalence divided by MDA tertiles NEAP quartiles and PRAL quartiles. Data are expressed as percentage of metabolic syndrome prevalence and analyzed using Fisher's exact test. *p-value < 0.05. MDA, Mediterranean diet adherence; PRAL, potential renal acid load; NEAP, net-endogenous acid production; MetS, metabolic syndrome.

Mediterranean diet adherence tertiles and PRAL quartiles were also used to evaluate the impact of the MDA and DAL on MetS prevalence and MetS score (Figure 1, Supplementary Tables 3A,B). MetS prevalence was not significantly affected by MDA, with a similar trend being observed for the MetS score. Instead, individuals in the strong PRAL quartile had a higher prevalence of the MetS, which is explained by an increase in the number of participants with positive criteria for MetS diagnosis, including blood pressure, triglycerides, and fasting glucose. Similar results were obtained by stratifying the study participants according to NEAP values.

### CV Risk Score

The effect of eating habits on CV risk was evaluated by considering 3 different calculation risk tools (Table 5, Supplementary Table 4). As described for MetS, MDA did not affect the CV risk score independently of the algorithm used. On the contrary, the strong PRAL group had significantly higher ASCVD and CUORE scores relative to alkaline PRAL. The European SCORE (ESC) risk score was also higher in participants in the strong PRAL quartile, but it did not reach significance (*p* = 0.067). Higher NEAP was associated with higher ASCVD and CUORE scores (Supplementary Table 4).

**Table 5:** Cardiovascular risk scores per MDA score tertile and PRAL quartile.

	**Low-MDA**	**Medium-MDA**	**High-MDA**	**#*p*-value**	**§*p*-value**	**Alkaline-PRAL**	**Neutral-PRAL**	**Light-PRAL**	**Strong-PRAL**	**#*p*-value**	**§*p*-value**
**ASCVD Risk Score**	11.5 (3.1–31.6)	12.5 (2.8–35.9)	12.0 (2.9–35.0)	0.734	0.696	9.5 (5.3–16.3)	11.9 (6.8–19.7)	12.2 (6.4–18.3)	13.4 (7.4–21.3)	0.061	0.01^*^
**European SCORE**	3.4 (1.1–13.3)	4.3 (1.1–15.6)	3.9 (1.0–14.9)	0.461	0.482	3.1 (2.0–5.9)	3.9 (2.4–6.4)	3.7 (2.2–6.0)	4.4 (2.5–6.8)	0.297	0.067
**Cuore Risk Score**	5.0 (1.5–19.3)	5.6 (1.2–16.4)	4.5 (1.1–19.4)	0.203	0.138	3.6 (2.2–7.6)	5.1 (2.4–10.3)	5.1 (2.4–10.7)	7.3 (3.2–11.5)	0.01^*^	0.01^*^

*Data are expressed as median (95% CI). ^#^p-value is based on Kruskal–Wallis test to compare the MDA or PRAL subgroups. ^§^p-value is based on Mann–Whitney test to compare high MDA against low MDA or strong PRAL against alkaline PRAL. MDA, Mediterranean diet adherence; PRAL, potential renal acid load; ASCVD, atherosclerotic cardiovascular disease; SCORE, Systematic COronary Risk Evaluation*.

* *p-value < 0.05*.

## Discussion

This study aimed at shedding light on the relationship between MetS, estimated CV risk, and dietary habits, with a particular focus on MDA and DAL.

The results reported in this study indicate that the MDA score negatively correlates with DAL. Nonetheless, despite MDA being associated with an improvement in some metabolic health parameters, a higher MDA score was not concomitant with a lower prevalence of MetS or a decrease in CV risk, an effect which was observed in the study participants consuming diets with a low DAL. The statistical analyses have generally shown similar results for PRAL and NEAP, but with a higher statistical power for PRAL.

At present, to our knowledge, no study has analyzed the relationship between MDA score, DAL, and cardiometabolic health, especially to test the possibility that some of the metabolic benefits of the MD may be dependent on its low DAL. However, Bullò and coworkers (31) partially addressed this matter by investigating the effects of a 1-year intervention with an MD supplemented with olive oil or nuts on bone health. The authors reported a decrease in PRAL and NEAP values in the participants eating an MD supplemented with olive oil and an increase in those consuming an MD integrated with nuts (31). This is in line with the findings of this study, in which a higher MDA score was associated with lower PRAL and NEAP and the fact that the study participants reported high olive oil and low nut consumption.

The health-promoting effects ascribed to the MD appear to be mediated, at least in part, by the high intake of antioxidants derived from fruits, olive oil, and vegetables, as well as the intake of oleic acid, dietary fiber, non-refined carbohydrates, and plant proteins (32). These key nutritional features are reflected in this study, as indicated by a positive association between MDA score and the intake of vitamins with antioxidant activity (vitamin A, vitamin C, and vitamin E), dietary fiber, and potassium. Instead, these nutrients negatively correlated with PRAL. Furthermore, while no association was observed relative to the MDA score, NEAP and PRAL were positively correlated with saturated fatty acids and cholesterol intake. In light of this, while antioxidants and dietary fibers may contribute to the cardiometabolic protection exerted by the MD (33, 34), saturated fatty acids and cholesterol represent key elements in the development of cardiometabolic diseases (35). However, the MDA score did not correlate with saturated (negatively) and monounsaturated (positively) fatty acids. This may be explained by the fact that none of the study participants completely adhered to the MD, as witnessed by MDA scores ranging between 18.0 and 22.3 out of 26.

As already described by the EPIC-PANACEA project (36), the results presented in this study confirm the positive impact of the MD on body weight regulation. Study participants with a high-MDA score had a lower BMI and fat mass compared to participants in the low-MDA tertile. Instead, there were no differences in BMI and fat mass between participants stratified by PRAL values, although there were significant differences in waist circumference, free fat mass, total body water, muscle mass, and body cell mass between these groups. Findings described in the literature about the associations between DAL and BMI are controversial. An umbrella review by Farhangi et al. (37) reported either a positive, negative or no association between DAL and BMI. After analyzing all the data, they concluded that significant differences were observed only when participants were stratified by sex; higher BMI was associated with higher PRAL in women and with higher NEAP in men (37). Nevertheless, BMI is not a sufficient predictor of cardiometabolic outcomes as it does not take into consideration body composition nor fat distribution. In fact, differences in BMI may also reflect variations in fat-free mass and muscle mass, which increase in parallel with DAL and may be dependent on the higher protein intake. On the contrary, waist circumference represents a more accurate predictor of cardiometabolic health compared to BMI alone (38). Not surprisingly, in fact, study participants in the highest PRAL quartile had higher waist circumference measures, which supports the potential association between a higher DAL and cardiometabolic diseases.

In agreement with a previous meta-analysis (39), the results reported in this study revealed a better metabolic profile in participants with a higher MDA score. This was underlined by a lower HOMA-IR, suggesting an increase in insulin sensitivity and an improvement in the circulating levels of HDL cholesterol and triglycerides, albeit the latter did not reach statistical significance. Despite this, a higher MDA score was not associated with a reduction in the criteria for the diagnosis of MetS or MetS prevalence. On the contrary, the percentage of study participants with positive MetS diagnostic criteria, namely, circulating glucose, triglycerides levels, and blood pressure were higher in the strong PRAL group vs. the alkaline PRAL group, supporting the possibility that a higher DAL may impair metabolic health (16). Similar results were obtained by analyzing NEAP quartiles and MetS prevalence. These results are in agreement with previous reports indicating similar associations between PRAL as well as NEAP and the prevalence of MetS in Iranian patients with type 2 diabetes (40) and a cross-sectional Japanese study, in which higher NEAP values (PRAL was not calculated) were associated with an increased prevalence of MetS independently of sex, age, and BMI (17). Contradictory results were described by Jafari et al. (41) who observed an association between MetS prevalence and NEAP but not PRAL in a cohort of Iranian men. Instead, the other two Iranian studies reported no association between PRAL or NEAP and the MetS (42, 43). Diverging results may be due to differences in dietary quality and/or genetic, sociodemographic, and behavioral characteristics of the population. Furthermore, the lack of association between MDA and decrease in MetS prevalence may be dependent on the fact that the diet of the study cohort, including for the individuals with a higher MDA score, was not always strictly in line with the MD pyramid, as already described.

In this study, higher PRAL and NEAP values were associated with a heightened CV risk regardless of the risk score used. Similar results were reported in the only study conducted to date (44). Regarding the MDA score, instead, no association with CV risk was observed. However, this is surprising, especially considering this dietary pattern being widely demonstrated to exert protective effects against major CV events (7). The reason for this discrepancy may be dependent on the fact that this study was not a strict dietary intervention, but it is based on food frequency questionnaires and by the fact that the recommendations set by the MD pyramid were not always followed by a large portion of the study population, as in the case of red meat consumption. Furthermore, while a Mediterranean diet supplemented with nuts has previously been shown to increase DAL compared to an MD supplemented with olive oil (31), it still exerted a protective effect against CV events (7). This suggests that despite DAL being associated with CV risk, alone it is not sufficient to explain CV risk. Instead, CV risk is more likely to depend upon overall diet quality. In fact, in this study, a higher DAL was positively associated with the intake of total lipids, saturated fatty acids, cholesterol, and sodium, with all these nutrients being linked with an increase in CV risk (45). Thus, in this study, a low DAL may represent an indicator of healthier dietary choices linked with better cardiometabolic health. This is in agreement with the metabolically detrimental effects of the Western diet, which, in fact, is a highly acidogenic dietary pattern (16, 46).

The results described here allow various reflections. First, it is possible to state that adherence to the MD, measured as the frequency of food consumption without considering the number of nutrients ingested, does not reflect MetS prevalence nor CV risk. An increase in the predictive power could be given by considering not only the diet but also other parameters associated with the Mediterranean lifestyle, such as culinary, social, and physical activity habits. An example of this is the MEDLIFE index, developed by Sotos-Prieto et al. (47) which includes fifteen items relative to food consumption; seven items about traditional Mediterranean dietary habits; and six items about physical activity, rest, and social interactions.

Second, especially when assessed by PRAL, DAL was associated with worse indices of metabolic and CV prognosis. Positive linear associations of PRAL and pathological conditions are described in literature except in a studio by Xu et al. (48) in which a modest non-linear U-shaped relation between mortality rates and PRAL was found, with a worse prognosis for both dietary acid and alkali excess. However, in this study, alkaline PRAL values are lower than values observed in this study (minimal values of PRAL were −111 and −41.9 mEq/day, respectively). Therefore, the present results could not exclude potential negative effects of alkali excess in the diet.

This study has several strengths. First, the MDA score, PRAL, and NEAP were estimated based on questionnaires and algorithms administered or calculated by trained interviewers and not self-administered questionnaires as reported in other studies. Second, DAL was assessed by two tools (PRAL and NEAP), while the CV risk score was calculated by 3 different validated algorithms, and the results were similar. Third, participants comprised a relatively large and well-characterized population. Fourth, to the best of our knowledge, this was the first study to investigate the relationship between indices of metabolic and CV health and dietary parameters, namely MDA score and DAL.

This study also presents some limitations, which need to be taken into consideration. First, the lack of consensus on how to assess MDA made it difficult to compare the present results with previously published studies reporting on MDA scores. In fact, there are almost 30 scores based on different food frequency questionnaires to assess MDA (49). PRAL and NEAP are calculated by a mathematical equation using levels of nutrients estimated with software (Winfood) using the average of the two 24-h recalls.

## Conclusion

There is no doubt that the MD pattern is associated with lower CV risk, but the MDA score calculated using food frequency did not correlate with metabolic and CV state defined as the prevalence of MetS and estimated CV risk. Instead, DAL values, especially those assessed using PRAL computed from 24-h recalls, more closely relate to a cardiometabolic health state.

## Data Availability Statement

The raw data supporting the conclusions of this article will be made available by the authors upon reasonable request.

## Ethics Statement

The study involving human participants was approved by National Ethical Committee of the Slovenian Ministry of Health and reviewed and approved by Comitato Etico di Area Vasta Emilia Centro (CE-AVEC). The participants provided their written informed consent to participate in this study.

## Author Contributions

JS and AP: design of the study and data analysis. JS, SC, EC, RS, and FGDG: acquisition of data. JS, DS, and AP: data interpretation. JS and DS: drafting of the article. GB, SL, BS, RP, and AP: critical revision of the article. All authors read and approved the final manuscript.

## Funding

This study was a part of the research project Physical Activity and Nutrition for Quality Aging (PANGeA), supported by a grant from the Cross-border Cooperation Program Slovenia, Italy 2007–2013, grant number 042-2/2009.

## Conflict of Interest

The authors declare that the research was conducted in the absence of any commercial or financial relationships that could be construed as a potential conflict of interest.

## Publisher's Note

All claims expressed in this article are solely those of the authors and do not necessarily represent those of their affiliated organizations, or those of the publisher, the editors and the reviewers. Any product that may be evaluated in this article, or claim that may be made by its manufacturer, is not guaranteed or endorsed by the publisher.
