# Supplementary material for: Dietary Acid Load but Not Mediterranean Diet Adherence Score Is Associated With Metabolic and Cardiovascular Health State: A Population Observational Study From Northern Italy
Source: Front Nutr. 2022 Apr 26;9:828587. doi: 10.3389/fnut.2022.828587 (PMC9087734; doi:10.3389/fnut.2022.828587)
Supplement: Supplementary file 1 [file Data_Sheet_1.docx]

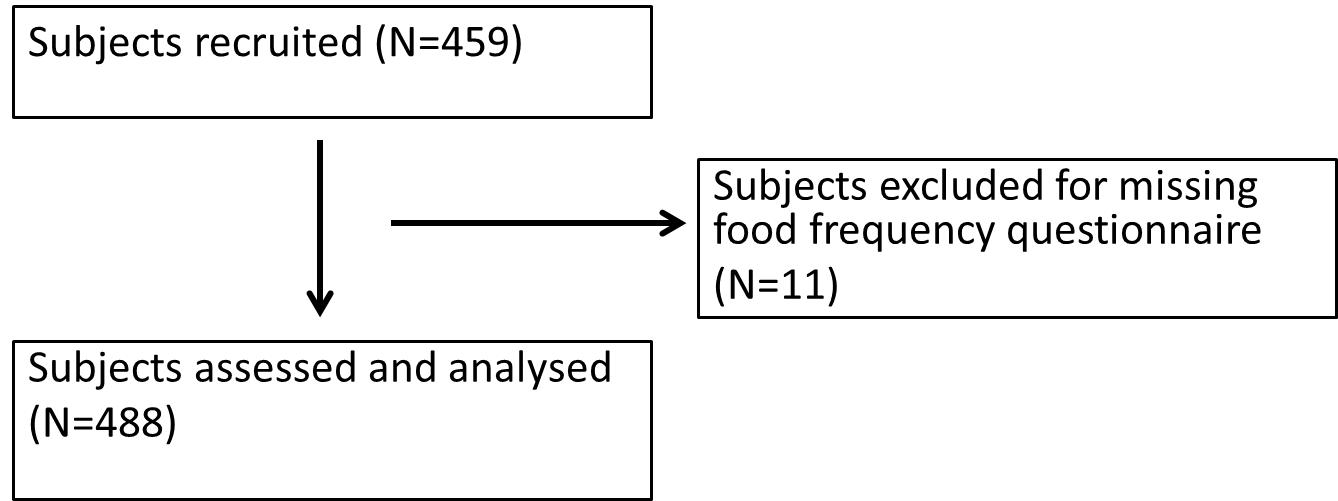


Figure 1S. Flowchart of participants.

Table S1. Scoring of the 13 main food categories for the calculation of the MDA score.

|  | Score 0 | Score 1 | Score 2 |
| --- | --- | --- | --- |
| **Milk and dairy products** | less than 4 times a week | 5/6 times a week | 1 or more times a day |
| **Fish** | 1 or more times a day or less than 4 times a month | once a week or 5/6 times a week | 2-4 times a week |
| **Egg** | more than 4 times a week or less than 4 times a month | 1 time a week | 2-4 times a week |
| **White meat** | more than 4 times a week or almost never | less than 4 times a month | 1-4 times a week |
| **Red meat and cold cuts** | more than 4 times a week or almost never | 2-4 times a week | 1-4 times a month |
| **Wine** | less than 5 times a month | more than 1 time a day or 2-6 times a week | 1 time a day |
| **Sweet desserts** | more than 4 times a week | 2-4 times a week | less than 5 times a month |
| **Olive oil** | less than 1 time a day | once a day | more than once a day |
| **Vegetables** | less than 5 times a week | 1 time a day or 5/6 times a week | more than once a day |
| **Fruit** | less than 5 times a week | 1 time a day or 5/6 times a week | more than once a day |
| **Nuts and seeds** | less than 5 times a week | 5/6 times a week | 1 or more times a day |
| **Cereals and grain** | less than 5 times a week | 5-7 times a week | more times a day |
| **Legumes** | more than 4 times a week or less than 4 times a month | 1 time a week | 2-4 times a week |

Table S2. Percentage of compliance with the recommendations of the Mediterranean diet pyramid for the consumption of 13 main food categories.

|  | MDA score | | |
| --- | --- | --- | --- |
|  | Score 0 | Score 1 | Score 2 |
| **Sweet deserts (%)** | 68.1 | 16.5 | 15.4 |
| **Nuts and seeds (%)** | 73.0 | 7.6 | 19.4 |
| **Red and processed meat (%)** | 39.1 | 41.5 | 19.4 |
| **Wine (%)** | 33.9 | 42.4 | 23.7 |
| **Egg (%)** | 27.2 | 39.7 | 33.0 |
| **Legumes (%)** | 20.3 | 40.4 | 39.3 |
| **Fish (%)** | 20.5 | 36.8 | 42.6 |
| **White meat (%)** | 12.5 | 15.8 | 71.7 |
| **Olive oil (%)** | 4.9 | 18.1 | 77.0 |
| **Fruit (%)** | 5.6 | 10.5 | 83.9 |
| **Grain (%)** | 4.5 | 7.8 | 87.5 |
| **Milk and dairy (%)** | 7.1 | 4.9 | 87.9 |
| **Vegetables (%)** | 1.8 | 5.4 | 92.9 |

­­­­­­­Table S3A. Percentage of subjects with MetS or with positive criteria for diagnosis of MetS stratified by MDA score tertiles or PRAL quartiles.

|  | Low-MDA | Medium-MDA | High-MDA | #p-value | §p-value | Alkaline-PRAL | Neutral-PRAL | Light-PRAL | Strong-PRAL | #p-value | §p-value |
| --- | --- | --- | --- | --- | --- | --- | --- | --- | --- | --- | --- |
| **MetS, yes (%)** | 19.7 | 16.54 | 14.2 | 0.445 | 0.215 | 10.3 | 15.7 | 17.9 | 22.2 | 0.124 | 0.026* |
| **Waist circ., yes %)** | 48.53 | 38.4 | 45.7 | 0.217 | 0.647 | 43.2 | 40.7 | 50.9 | 42.9 | 0.439 | 1 |
| **BP, yes (%)** | 81.6 | 81.9 | 72.8 | 0.083 | 0.079 | 73.0 | 78.8 | 76.8 | 84.8 | 0.185 | 0.034* |
| **TG, yes (%)** | 14.7 | 8.2 | 10.4 | 0.19 | 0.294 | 5.6 | 11.1 | 11.7 | 16.8 | 0.081 | 0.016* |
| **HDL-C, yes (%)** | 8.1 | 5.1 | 6.4 | 0.616 | 0.658 | 3.7 | 7.4 | 10.9 | 4.6 | 0.139 | 1 |
| **Glucose, yes (%)** | 19.1 | 20.3 | 18.5 | 0.957 | 1 | 13.1 | 17.6 | 19.6 | 27.5 | 0.057 | 0.011* |
| Data is expressed as percentage of subject with MetS or positive criteria for MetS. #p-value is based on Chi-squared tests to compare the MDA or PRAL subgroups. §p-value is based on Fisher's exact test to compare High-MDA against Low-MDA or Strong-PRAL against Alkaline-PRAL. MDA, Mediterranean diet adherence; PRAL, potential renal acid load; MetS, Metabolic syndrome; Waist circ., waist circumference; BP, Blood pressure; TG, triglycerides; HDL-C, high-density lipoprotein cholesterol. | | | | | | | | | | | |

Table S3B. Percentage of subjects with MetS or with positive criteria for diagnosis of MetS stratified by NEAP quartiles.

|  | Q1-NEAP | Q2-NEAP | Q3-NEAP | Q4-NEAP | #p-value | §p-value |
| --- | --- | --- | --- | --- | --- | --- |
| **MetS, yes (%)** | 11.2 | 15.6 | 17.3 | 22.2 | 0.185 | 0.044* |
| **Waist circ., yes (%)** | 42.0 | 40.2 | 48.2 | 47.3 | 0.473 | 0.441 |
| **BP, yes (%)** | 70.5 | 77.7 | 78.6 | 86.6 | 0.064 | 0.008** |
| **TG, yes (%)** | 7.1 | 8.9 | 11.6 | 16.1 | 0.134 | 0.058 |
| **HDL-C, yes (%)** | 4.5 | 7.1 | 7.1 | 7.1 | 0.814 | 0.569 |
| **Glucose, yes (%)** | 10.7 | 22.3 | 19.6 | 23.2 | 0.072 | 0.019* |
| Data is expressed as percentage of subject with MetS or positive criteria for MetS. # p-value is based on Chi-squared tests to compare the NEAP subgroups. § p-value is based on Fisher's exact test to compare Q1-NEAP against Q4-NEAP. NEAP, net-endogenous acid production; MetS, Metabolic syndrome; Waist circ., waist circumference; BP, Blood pressure; TG, triglycerides; HDL-C, high-density lipoprotein cholesterol. | | | | | | |

Table 4S. Cardiovascular Risk Scores per NEAP-quartile.

|  | Q1-NEAP | Q2-NEAP | Q3-NEAP | Q4-NEAP | #p-value | §p-value |
| --- | --- | --- | --- | --- | --- | --- |
| **ASCVD Risk Score** | 9,9 (2,9 - 34,2) | 12,0 (3,5 - 31,4) | 12,7 (2,7 - 35,8) | 12,8 (2,9 - 36,6) | 0.138 | 0.053 |
| **European SCORE** | 3,2 (1,0 - 14,9) | 3,9 (1,2 - 13,3) | 3,7 (1,1 - 15,4) | 4,7 (1,0 - 15,0) | 0.383 | 0.103 |
| **Cuore Risk Score** | 3,7 (1,1 - 14,7) | 5,8 (1,5 - 20,9) | 4,9 (1,2 - 19,9) | 5,6 (1,2 - 17,9) | 0.036* | 0.017* |
| Data is expressed as median (95% CI). #p-value is based on Kruskall-Wallis test to compare the NEAP quartiles. §p-value is based on Man-Whitney Test to compare Q4-NEAP against Q1-NEAP. NEAP, net-endogenous acid production; ASCVD, AtheroSclerotic CardioVascular Disease; SCORE, Systematic COronary Risk Evaluation. | | | | | | |

Table S5. Correlation analysis between MDA, PRAL or NEAP and Cardiovascular Risk Scores.

|  | MDA | PRAL (mEq/day) | NEAP (mEq/day) |
| --- | --- | --- | --- |
|  | *r_S_* | *r_S_* | *r_S_* |
| **ASCVD Risk** | 0.013 | 0.137** | 0.103* |
| **European SCORE** | 0.023 | 0.102* | 0.083 |
| **Cuore Score Risk** | -0.103 | 0.180** | 0.120* |
| MDA, Mediterranean diet adherence; PRAL, potential renal acid load; NEAP, net-endogenous acid production; r_S:_ Spearman’s correlation coefficient; ASCVD, AtheroSclerotic CardioVascular Disease; SCORE, Systematic COronary Risk Evaluation. * p-value <0.05, ** p-value <0.01. | | | |
